# PAPreC: A Pipeline for Antigenicity Prediction Comparison Methods across Bacteria

**DOI:** 10.1021/acsomega.4c07147

**Published:** 2025-02-03

**Authors:** Yasmmin C. Martins, Maiana O. Cerqueira e Costa, Miranda C. Palumbo, Dario F. Do Porto, Fábio L. Custódio, Raphael Trevizani, Marisa Fabiana Nicolás

**Affiliations:** † Bioinformatics Laboratory, National Laboratory for Scientific Computing, Av. Getúlio Vargas 333, 25651-075 Petrópolis, Brazil; ‡ Department of Biological Chemistry, Faculty of Exact and Natural Sciences, University of Buenos Aires - UBA, Av. Int. Cantilo, C1428 Buenos Aires, Argentina; ¶ Department of Computational Mechanics, National Laboratory for Scientific Computing, Av. Getúlio Vargas 333, 25651-075 Petrópolis, Brazil; § Biotechnology, Oswaldo Cruz Foundation - Fiocruz, Street São José S/N, 61760-000 Eusébio, Brazil

## Abstract

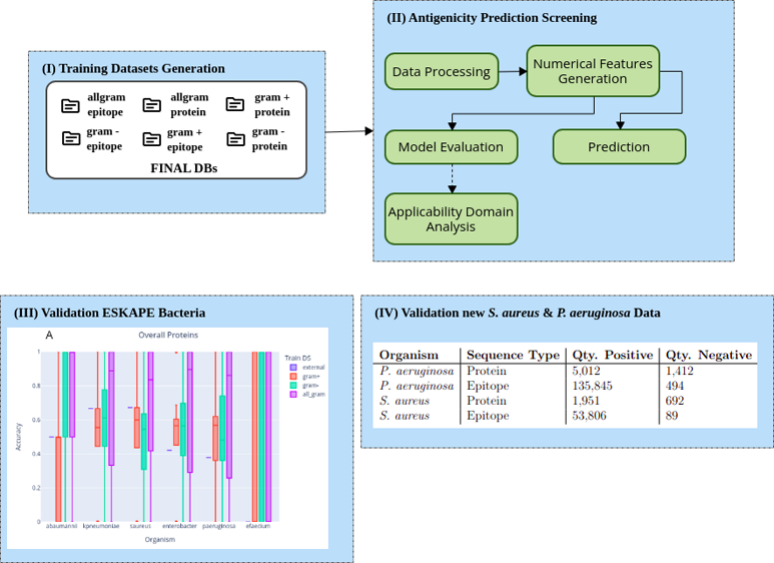

Antigenicity prediction plays a crucial role in vaccine development, antibody-based therapies, and diagnostic assays, as this predictive approach helps assess the potential of molecular structures to induce and recruit immune cells and drive antibody production. Several existing prediction methods, which target complete proteins and epitopes identified through reverse vaccinology, face limitations regarding input data constraints, feature extraction strategies, and insufficient flexibility for model evaluation and interpretation. This work presents PAPreC (Pipeline for Antigenicity Prediction Comparison), an open-source, versatile workflow (available at https://github.com/YasCoMa/paprec_nx_workflow) designed to address these challenges. PAPreC systematically examines three key factors: the selection of training data sets, feature extraction methods (including physicochemical descriptors and ESM-2 encoder-derived embeddings), and diverse classifiers. It provides automated model evaluation, interpretability through SHapley Additive exPlanations (SHAP) analysis, and applicability domain assessments, enabling researchers to identify optimal configurations for their specific data sets. Applying PAPreC to IEDB data as a reference, we demonstrate its effectiveness across the ESKAPE pathogen group. A case study involving **Pseudomonas aeruginosa** and *Staphylococcus aureus* shows that specific feature configurations are more suitable for different sequence types, and that ESM-2 embeddings enhance model performance. Moreover, our results indicate that separate models for Gram-positive and Gram-negative bacteria are not required. PAPreC offers a comprehensive, adaptable, and robust framework to streamline and improve antigenicity prediction for diverse bacterial data sets.

## 1 Introduction

Antigenicity refers to the ability of a molecule, termed an antigen, to elicit an immune response through specific binding to immune system receptors. Such responses can be protective—characterized by antibody production and/or T-cell activation (immunogenicity)—or deleterious, as in allergic reactions.^1^ Understanding antigenicity requires the identification of epitopes, the immunogenic region of an antigen that is specifically recognized by the immune system components B and T cells to induce humoral and cellular immune responses, respectively.^2^ B cells recognize antigenic determinants (B-cell epitopes, BCEs), which may be linear or conformational depending on whether recognition depends on the amino acid sequence or in the three-dimensional structure of the protein.^3^ T-cell epitopes (TCEs) are fragments of sequences derived from protein antigens present on antigen-presenting cells (APCs) via major histocompatibility complex (MHC) molecules and are recognized by specific T-cell subtypes. CD8+ T cells, known as cytotoxic T lymphocytes (CTLs), are activated through interaction with MHC class I molecules. In contrast, CD4+ T cells, denominated as helper T lymphocytes, interact via MHC class II molecules.^4^ Antigens can be categorized into various subclasses, among which protective antigens.

Vaccination, the most effective method for disease prevention and mortality reduction, can be formulated using either attenuated pathogens or purified subunits containing complete or partial protective antigens. Conventional vaccine development faces significant challenges, including the inability to cultivate several pathogens in laboratory conditions and identifying antigens exclusively expressed in vitro. The limitations of the laborious antigen identification process associated with the advent of high-throughput sequencing technology enabled the emergence of Reverse Vaccinology (RV).^5^ The RV relies on genomic data and bioinformatics tools to identify critical features within protein sequences, leading the selection of potential vaccine candidates toward experimental validation. This approach reduces both the time and cost compared to the traditional method of vaccine development.^2^ Reverse vaccinology was first successfully applied to identify vaccine candidates against serogroup B *Neisseria meningitidis*, a significant cause of meningococcal meningitis, when Pizza et al. discovered 25 immunogenic proteins, five of which were incorporated into the final formulation of Bexsero, the first RV-derived vaccine approved for use (https://www.fda.gov/vaccines-blood-biologics/vaccines/bexsero).^6,7^ As antimicrobial resistance escalates, vaccines become even more critical, potentially reducing antibiotic use and decreasing the spread of bacterial resistance.^8^ Multiepitope vaccine strategies further enhance protection by simultaneously targeting multiple antigenic determinants.^9^

Bioinformatics-driven vaccine design has greatly benefited from databases compiling immunological data. For instance, Protegen (https://violinet.org/protegen/index.php) provides information about validated protective antigens from bacteria, viruses, and noninfectious agents like allergens and cancer antigens.^10^ The Immune Epitope Database (IEDB, https://www.iedb.org/) provides extensive experimental data on T- and B-cell epitopes related to various conditions, including infectious diseases and allergies, along with analysis tools for epitope prediction and evaluation.^11^ Together, these resources accelerate research in RV and personalized immunotherapies.

The first RV-oriented antigen discovery program, NERVE,^12^ represented a decision-tree or “filtering” approach, applying sequential criteria to select potential vaccine candidates. Subsequent tools introduced machine learning (“classifying”) strategies for antigenicity prediction, integrating measured or predicted features of proteins into numeric representations for classification.^13^ VaxiJen pioneered the application of alignment-free machine learning methods based on autocovariance of amino acid properties and neural networks to distinguish antigens from nonantigens in bacteria, viruses, and tumors.^14^ Vaxign-ML integrated biological and physicochemical data, implemented resampling techniques to handle imbalanced data sets, and performed feature selection and hyperparameter tuning on multiple classifiers to improve predictive robustness.^15^ Despite these advances, the accuracy and generalization of antigenicity predictors depend strongly on factors such as the composition and origin of training data sets, feature extraction methods, and classifier choices.^16,17^ A more systematic assessment of these elements is needed to refine prediction models and improve their performance and interpretability.

Meanwhile, recent approaches^18−20^ have shown that modeling peptides and proteins as graphs—mainly when supported by 3D structural data from AlphaFold or ESMFold—can capture subtle structural relationships and potentially strengthen predictive power. Such deep learning methods, however, often demand high-performance computing infrastructures and specialized GPU resources, which may limit their widespread applicability.

In this context, we present PAPreC (Pipeline for Antigenicity Prediction Comparison), a comprehensive pipeline designed to evaluate antigenicity predictions exploring key factors influencing predictive performance (available at https://github.com/YasCoMa/paprec_nx_workflow). While our pipeline was initially applied and tested for bacterial genomes, its modular design can be adapted for viruses, cancer antigens, and other models. PAPreC performs exhaustive evaluations by systematically modifying three key factors: (1) the source data set used for training, encompassing both short peptides and complete protein sequences; (2) the feature extraction methods employed, which incorporate three alignment-free methods, including ESM-2 encoder-derived embeddings^21^ and the other two apply covariance matrices in conjunction with physicochemical descriptors derived from the amino acid sequence; and (3) a varied set of experimental configurations comprising diverse classifiers. PAPreC allows transparent control of each step, offering SHapley Additive exPlanations (SHAP) for interpretability^22^ and applicability domain analyses to assess model reliability and robustness. Using Gram-positive and Gram-negative bacteria as a baseline, we evaluated PAPreC with IEDB reference data for the clinically significant ESKAPE group,^23^ showing promising results across key performance metrics. Finally, to illustrate practical usefulness, we applied PAPreC to predict epitopes from **Pseudomonas aeruginosa** and *Staphylococcus aureus* as a case study, demonstrating its adaptability and potential impact on future vaccine research and therapeutic strategies.

## 2 Materials and Methods

### 2.1 Training Data Sets Construction

We selected three primary data sets (Table 1) following criteria established in recent antigenicity prediction methods^15,24^—the first data set, Bcipep (Saha et al.), initially comprised 2484 epitopes. After removing sequences with nonstandard amino acid symbols, we obtained 1011 positive and 204 negative cases. We treated this data set independently, as T- and B-cell targets rely on distinct recognition mechanisms.^25^

**Table 1:** Description of the Datasets Employed along with Their Processing Approaches

data set	type	composition	size	year	data preparation
Bcipep	B-cell epitopes	positive and negative	2484 peptides	2005	filtered out peptides with nonstandard amino acid symbols
HLA	T-cell epitopes	positive and negative	6771 peptides	2018	no additional processing required
Protegen	protective antigens	positive	1631 sequences	2011	augmented and generated negative cases using UniProt proteomes and the IEDB database

**Figure 1:**
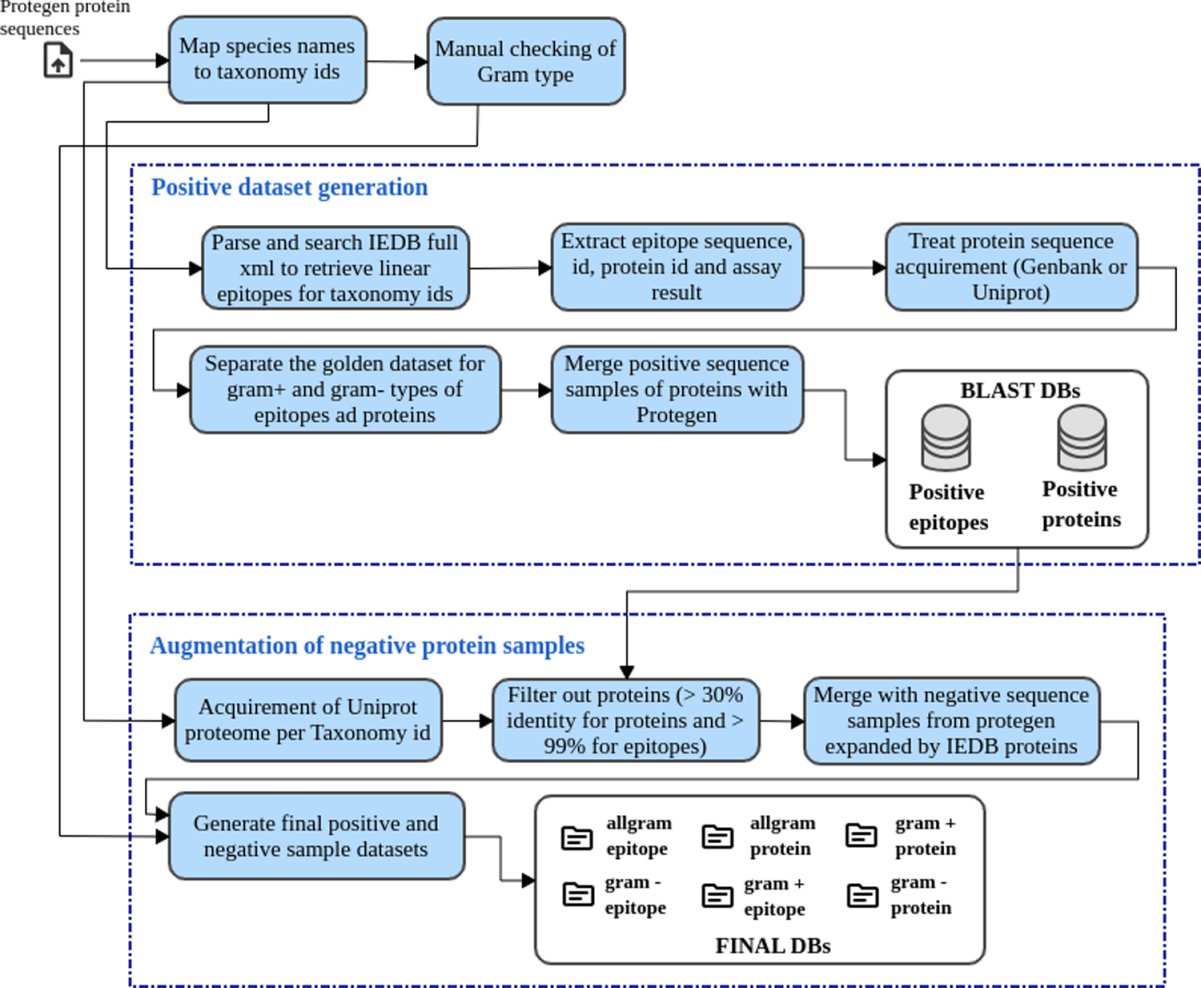
Diagram illustrating the positive and negative sequence generation through augmentation from IEDB epitope information retrieved according to the mapped taxonomy identifiers of species present in Protegen sequences.

The second data set, HLA,^26^ required no additional preprocessing. The third data set, Protegen,^10^ contained only positive bacterial protein sequences, necessitating negative sample generation. We retrieved the corresponding proteomes for each bacterium from UniProt and followed the procedure in Figure 1 to integrate negative cases. Four augmented data sets—covering epitopes and proteins from both Gram-positive (14 species) and Gram-negative (36 species) bacteria—were then constructed.

To update and expand Protegen’s positive protein sequences, we integrated data from IEDB, which frequently updates its epitope annotations. After merging the Protegen proteins with IEDB-derived positive sequences, we generated two local BLAST databases. We identified negative proteins as those with less than 30% identity to the Protegen gold-standard sequences.^15^ Subsequently, we applied a second filter to ensure no candidate negative sequence shared more than 99% identity with any positive epitope. Sequences passing both criteria were accepted as negatives. This approach yielded comprehensive training data sets for model screening and evaluation under various conditions.

### 2.2 Pipeline for Antigenicity Prediction Comparison (PAPreC)

**Figure 2:**
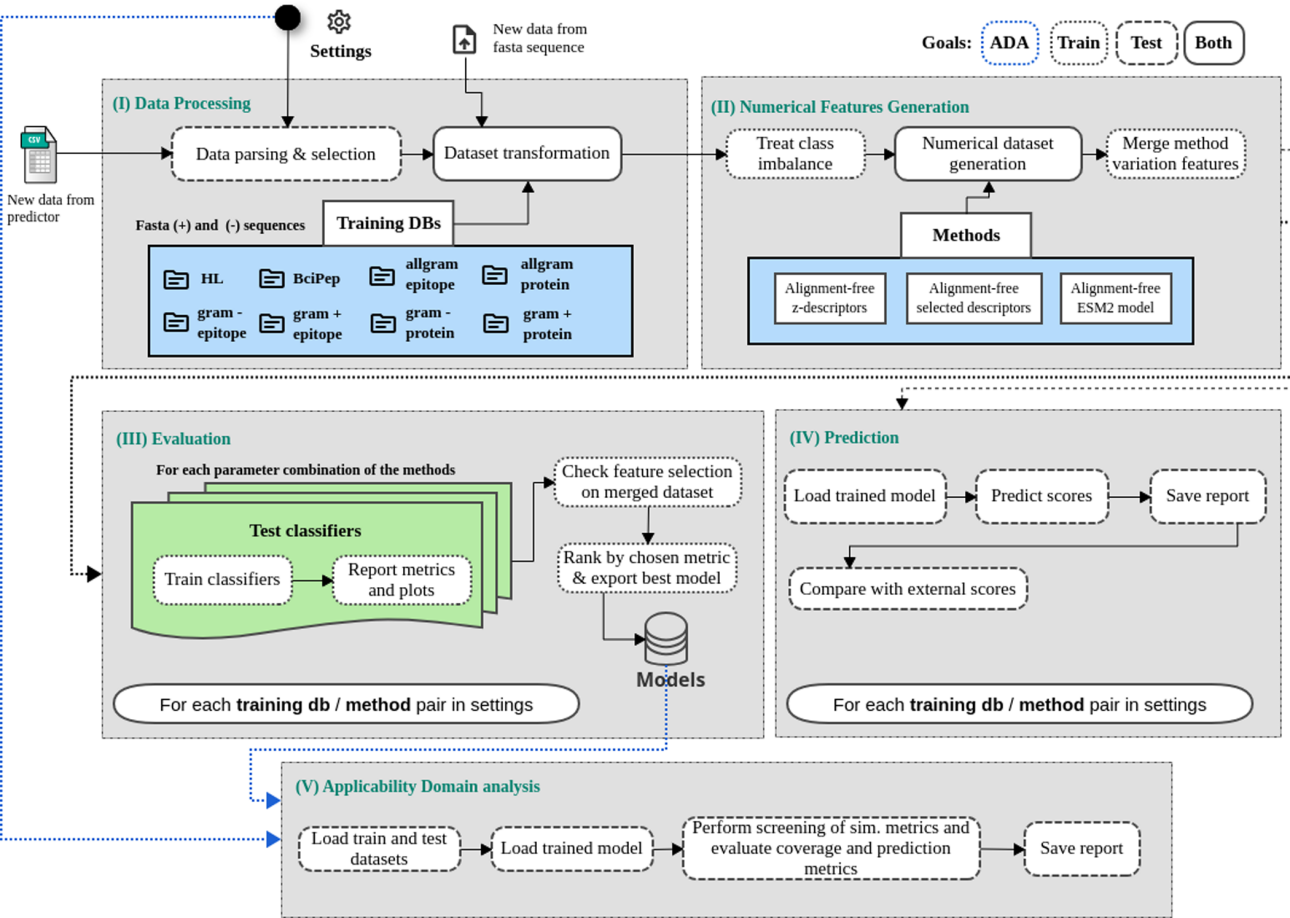
Diagram illustrating the sequential steps undertaken by PAPreC for: (i) data set preprocessing, (ii) numerical feature generation, (iii) evaluation, and (iv) prediction. As input, the workflow receives a configuration file with details about thresholds for data filtering, new data set location, data sets, and feature extraction methods for training or test mode. In (i), PAPreC extracts each data set source and prepares the raw data into their respective positive and negative classes, standardizing the input for the next step. Step (ii) treats the balancing of the positive and negative sample data according to the technique specified by the user. Also, it calculates the numerical feature matrices using the two alignment-free methods (described in Section 2.2.2) based on the covariance matrix of AA descriptors and the third method based on the embeddings from the ESM-2 autoencoder model. In this step, the features created from the methods’ variations are merged into one tabular data set for feature selection. Step (iii) In training mode, it follows a 10-fold model training for each feature subset from the method variations (auto or cross mode and the lag length, when applicable). The pipeline for the evaluation consists of fitting the model splitting in 10-fold cross-validation, getting the metric scores for the distinct classifiers, and ranking the models in two levels: classifier and method variation. At the end of this ranking process for each training data set and method combination, only one model is saved and used as a representative. Step (iv) is performed in the test task mode to predict the scores according to the best models elected in the training phase. It is possible to configure a reference table to compare the predictions to an external tool or database. Finally, step (v) is performed in the ADA (Applicability Domain Analysis) task mode. It executes a screening of the models coming from a specific target (sequence type, being either protein or epitope) to check if the predictions agree with the neighborhood expected and the similarity of test and train samples.

We developed PAPreC (Figure 2) as an automated workflow to evaluate antigenicity prediction models under varying conditions. The pipeline systematically adjusts three key factors based on established approaches^27−29^: (1) the source training data set (such as Bcipep, HLA,^26^ and Protegen^10^); (2) alignment-free methods for numerical feature generation; and (3) multiple classifier configurations. Although initially demonstrated with bacterial epitopes, PAPreC’s design facilitates adaptation to other organisms.

#### 2.2.1 Data Processing

The input sequences can be FASTA files (training and test mode) or raw NetMHCPanII outputs (only test mode), depending on their source. From the raw NetMHCPanII output, the pipeline filters epitopes by thresholds of binding affinity to MHC alleles, allele promiscuity, and sequence similarity in relation to positive epitopes database (this threshold is deactivated passing a valor above 1), ensuring only novel epitopes move forward (in case the similarity threshold is set up as 100%). These thresholds can be customized by the user following the instructions found in PAPreC GitHub repository. Similarity checks employ the Levenshtein metric,^30^ and human homology is assessed using UniProt.^31^ Protegen data augmentation integrates additional sequences from IEDB,^11^ following the procedure outlined in Figure 1. The resulting data sets are stored for subsequent modeling steps.

#### 2.2.2 Numerical Features Generation

PAPreC applies three alignment-free methods to extract numerical features. One method uses ESM-2 embeddings,^21^ truncating sequences to 2048 residues.^32,33^ The architecture employed in this study was the lightest ESM-2 variant, comprising six layers and approximately 8 million parameters.

The other two methods derive from auto- and cross-covariance matrices,^15,34,35^ incorporating amino acid physicochemical descriptors (e-descriptors and selected AAindex features).^36,37^ Lag values range from 1 to 8, optimizing the encoding for sequence length. PAPreC organizes and indexes the resulting features, enabling downstream feature selection and SHAP analysis^38^ to identify globally important attributes and support the interpretability analysis.

#### 2.2.3 Evaluation

The workflow employs PyCaret^39^ to train and evaluate models with various classifiers (Extra Trees, Random Forest, AdaBoost, Gradient Boosting classifier, Decision Tree, Support Vector Machine, Light and Extreme Gradient Boosting, Naive Bayes, and CatBoost). It automatically selects the best model per configuration based on accuracy, precision, recall, F1-score, area under the receiver operating characteristic curve (ROC-AUC), Cohen’s kappa, and Matthews correlation coefficient (MCC). PAPreC also generates ROC curves, precision-recall plots, confusion matrices, and SHAP plots for models using combined feature sets.

#### 2.2.4 Prediction

In test mode, PAPreC uses the previously selected best-performing models to predict probabilities and classes for new data sets. If a comparison file with labels is provided, the pipeline computes performance metrics for these predictions, summarizing the model’s effectiveness on novel input.

#### 2.2.5 Applicability Domain Analysis

To assess model reliability, PAPreC incorporates K-nearest neighbors analysis,^40^ evaluating how test samples relate to training data distributions. This analysis, using distance metrics such as Euclidean and cosine,^41^ identifies inliers and calculates performance metrics restricted to these subsets. By examining coverage and neighbor recovery, PAPreC provides insights into the accuracy of model predictions beyond global metrics.

### 2.3 Application in Experimental Curated Data

We applied the top-ranked PAPreC models to test sets derived from epitopes validated by published assays. These epitopes and their corresponding proteins were retrieved from the IEDB database (https://www.iedb.org) using data exported on September 17, 2024. Our analysis focused on the ESKAPE pathogen group due to their known for its significant antimicrobial resistance.^23^ We selected linear epitopes in the IEDB using the specific taxonomy identifiers for each of the six ESKAPE species: *Enterococcus faecium*, *Staphylococcus aureus*, **Klebsiella pneumoniae**, **Acinetobacter baumannii**, **Pseudomonas aeruginosa**, and *Enterobacter*. Nonrelevant taxa (for example, bacteriophages, *Serratia*, *Proteus*, and *Qubevirus durum*) were excluded. For each ESKAPE species, we compiled four data sets: all proteins, all epitopes, T-cell epitopes, and B-cell epitopes.

These data sets were evaluated using all relevant models produced in the training phase, using the produced data sets described in Section 2.1. To contextualize PAPreC’s performance, we compared its predictions with those from two established tools: VaxiJen for epitopes^14^ and Vaxign-ML for proteins.^15^

### 2.4 Application of PAPreC for New Epitope Discovery Case Study

We applied PAPreC to assess predicted antigenic epitopes from *P. aeruginosa* strain 14182 and *S. aureus* Bmb9393, both strains are clinically significant pathogens from the ESKAPE group. The *P. aeruginosa* 14182 strain, isolated in 2017 from a Brazilian tertiary hospital, carries a subclass B1 metallo-beta-lactamase gene (BioProject NCBI: PRJNA761695), while *S. aureus* subsp. *aureus* Bmb9393, isolated in 1993 in Rio de Janeiro,^42^ exhibits enhanced biofilm formation and the capacity to invade human airway cells. Their proteomes include 6518 proteins for *P. aeruginosa* and 2686 for *S. aureus*.

#### 2.4.1 Data Selection and Epitope Prediction

We targeted MHC class II epitopes using NetMHCPanII,^43^ testing 26 alleles widely represented in global populations^44^ and four additional alleles prevalent in Argentina and Brazil.^45^ The predicted epitopes were input for PAPreC in test mode, and default filtering thresholds from the “Data Parsing and Selection” step were applied.

#### 2.4.2 Prediction with PAPreC Models and Validation Strategies

We evaluated the best-ranked PAPreC-trained models—derived from all training data sets and feature extraction methods—against well-established tools for antigenicity prediction: VaxiJen v.2.0^14^ and Vaxign-ML.^15^ VaxiJen supports a range of pathogen types, while Vaxign-ML focuses on bacteria. We provided NetMHCPanII output directly to PAPreC and retrieved protein sequences from UniProt (UniProt Consortium 2019) to ensure consistent data inputs. VaxiJen with a threshold: of 0.7 was used at the epitope level and Vaxign-ML at the protein level, allowing direct comparison with PAPreC’s predictions.

To verify the recovery of known antigenic proteins and epitopes, we excluded epitopes initially present in the nonaugmented Protegen data set. We then assessed epitope and protein recovery in the augmented positive set using an 80% identity threshold for proteins and 100% for epitopes. Additionally, we examined Pfam domains to ensure that functional domains associated with bacterial immune responses remained present. By comparing PAPreC’s predictions with those from VaxiJen and Vaxign-ML, we evaluated its consistency and performance relative to experimentally validated IEDB data.

## 3 Results

### 3.1 Antigenicity Performance

Regarding the data selection to prepare the training data sets used to build the models for performance assessment, the final configuration of the positive and negative sample count on each data set is shown in Table 2. These data sets can be found in the PAPreC GitHub repository. The values acquired for the allgram protein (2098 positive and 174,671 – negative) and epitope (4826 positive and 25,535 – negative) correspond to the augmented data from Protegen. The full table with the IEDB-retrieved information enriched with the protein sequence, taxonomy identifier, and Gram type classification can be found in Supplementary Table 1.

**Table 2:** Number of Positive and Negative Samples in Each Prepared Training Dataset

data set/counts	positive	negative
hla	1032	5727
bcipep	1011	204
Gram-positive protein	1169	55,901
Gram-negative protein	929	118,853
Gram-positive epitope	2942	23,375
Gram-negative epitope	1884	2160
allgram protein	2098	174,671
allgram epitope	4826	25,535

**Figure 3:**
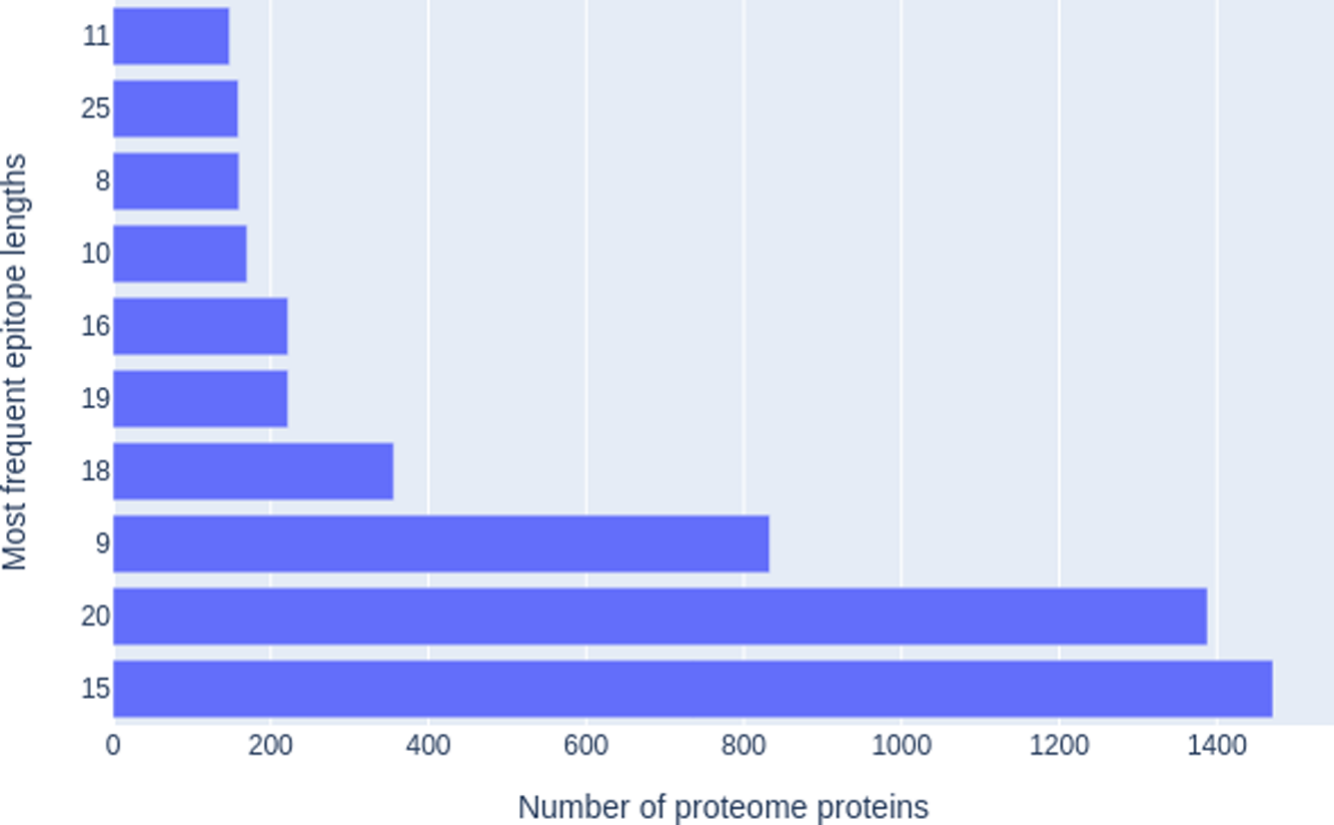
Frequency of retrieved epitopes by length. The figure illustrates the frequency of epitopes, categorized by their length, identified in the tested proteome sequences. Hit counts were obtained by aligning negative candidate sequences against a reference set of proteins and epitopes, with the results subsequently grouped according to epitope length.

When we selected negative protein sequences from the proteomes, we generated a quantification report (Supplementary Table 2) detailing the number of proteins and epitopes that matched our query sequences. In total, 163,090 proteins exhibited matches, among which 4545 proteins aligned with at least one positive epitope. As illustrated in Figure 3, epitopes ranging from 8 to 25 residues occurred with high frequency. For example, epitopes consisting of 15 and 20 amino acids were found in 1471 and 1388 proteins, respectively.

After preparing all training data sets, we used PAPreC in training mode to evaluate model performance across every combination of training data sets and alignment-free feature extraction methods. This process involved 24 pairs (8 training data sets combined with three feature extraction methods), executed in parallel on a standard computer using 5 out of 8 available cores and 16 GB of RAM. The feature generation step, which produced numerical representations, required 45 min and 39 s. The subsequent training and evaluation of the models for these combinations took approximately 3 h and 38 min.

The models generated for each combination of the training data set, alignment-free method, mode (auto or cross-variance), and lag (ranging from 1 to 8) corresponding to the method variations—except for the method based on ESM-2 autoencoder model embeddings^21^—were evaluated using 10-fold cross-validation, as implemented by default in PyCaret classification experiments.^39^ The Supplementary Table 3 reports the configuration that yielded the highest MCC values for the combinations. In Supplementary Figure 1, the overview of these results is stratified according to four relevant metrics: accuracy, ROC-AUC, F1, and MCC. The distribution of the values around the mean for each metric is only possible to perceive in the methods that allow variations (auto and cross-variance matrix and the lag lengths). The embedding-based values are represented by only one point. The models based on the protein sequence produce higher scores than the ones from the epitope training data sets, with mean values above 60% for accuracy, F1, and ROC-AUC in the Gram-positive and allgram in the feature extraction method based on the e-descriptors^36^ feature extraction method.

Supplementary Table 3 shows that, by ordering all the models without grouping by training database and feature extraction method, the top 20 ranked models contain representatives of all training data sets, with four presenting ROC-AUC above 80% and MCC above 45%. In 15 of these 20, the Extra Trees classifier ranked best among the tested classifiers. As perceived in the Supplementary Figure 1, these top 20 comprised mostly the ESM-2 model as a feature extraction strategy (present in 8 out of the top 10 models), followed by the mode variation concerning the feature selection-derived model, coming either from the AA Index or e-descriptors feature extraction methods. The Extra Trees is an ensemble classifier that internally combines many decision tree results to form a committee to return a consensus prediction, and this architecture is known to lead to models with enhanced accuracy and generalization.^46,47^ Furthermore, recent works also corroborate our findings concerning the good performance of models based on ESM-2 embeddings, outperforming the hand-crafted feature extraction methods by capturing more discriminative feature information.^48−50^

The MCC values are very low (below 0.5), with most mean values ranging from 0.1 to 0.3 across the combinations, denoting that the models have a poor agreement between the predicted and the true labels. However, the results of all these metrics are improved when applying the embeddings for the amino acid sequences generated from the ESM-2 model; the three models originated from whole protein sequences (gram-, gram+, and all gram) obtained an MCC above 0.4, and ROC-AUC values above 75%. This behavior was expected since this model was designed originally to represent whole protein sequences. The models from the epitope-based data sets training data set were also improved, surpassing 65% in the F1, ROC-AUC, and accuracy metrics.

**Figure 4:**
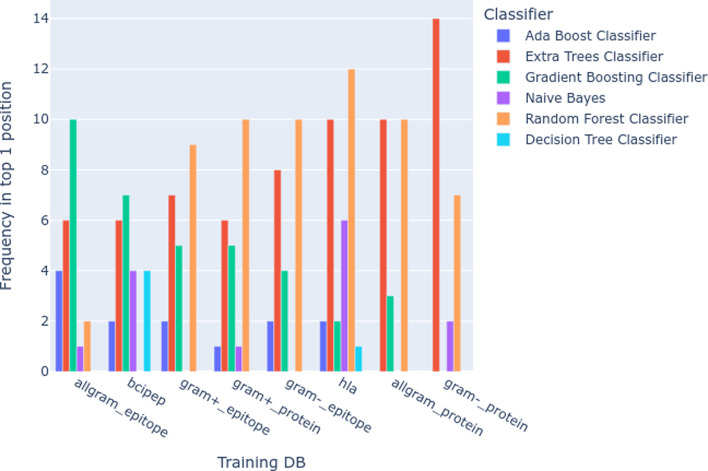
Frequency of each classifier as being the first among the tested models based on the MCC.

Regarding the analysis from the classifier perspective, the Extra Trees, followed by Gradient Boosting and Random Forest, were the top 3 relative to the best-ranked models listed by MCC, considering also the method variations. Interestingly, the Gradient boosting performed better in the epitope-based training data than in the protein-based one, as shown in Figure 4. Supplementary Figure 2 shows the values of the accuracy, ROC-AUC, F1, and MCC metrics for these classifiers stratified by the training data set. The mean values of these metrics for the Extra Trees classifier are very close to those observed for Random Forest, besides remaining in the 55–65% range. In both the Bcipep and Gram-negative epitope data sets, other classifiers—such as AdaBoost, Naive Bayes, and, in the case of Bcipep, Decision Tree—also ranked highly among the evaluated variations. Within the Bcipep data set, AdaBoost and Naive Bayes achieved comparable accuracy, and ROC-AUC values ranging from 59 to 65%.

Considering that feature selection produced the top-ranked models among the 24 tested combinations, we detailedly examined feature importance and reduction, leveraging PyCaret’s model selection and data transformation capabilities during the classification experiments. According to the results reported in Supplementary Table 4, The most important features across these models are achieved in the auto variance mode to calculate the matrices in the feature extraction methods based on e-descriptors and selected AAIndex descriptors. The feature importance analysis reveals no clear preference for the lag size in the amino acid neighborhood across the tested combinations, with lag values ranging from 1 (identified by l1) to a maximum of 8 (l8). The ranking position of the models based on feature selection, and the choice from distinct modes and sizes of lags suggest that the dimensionality and complexity reduction enhanced the performance from a global perspective. The feature selection process reduced the number of features from 200 to 40 for the e-descriptors strategy, while it decreased them from 48 to 10 in the selected AAIndex descriptors method.

**Figure 5:**
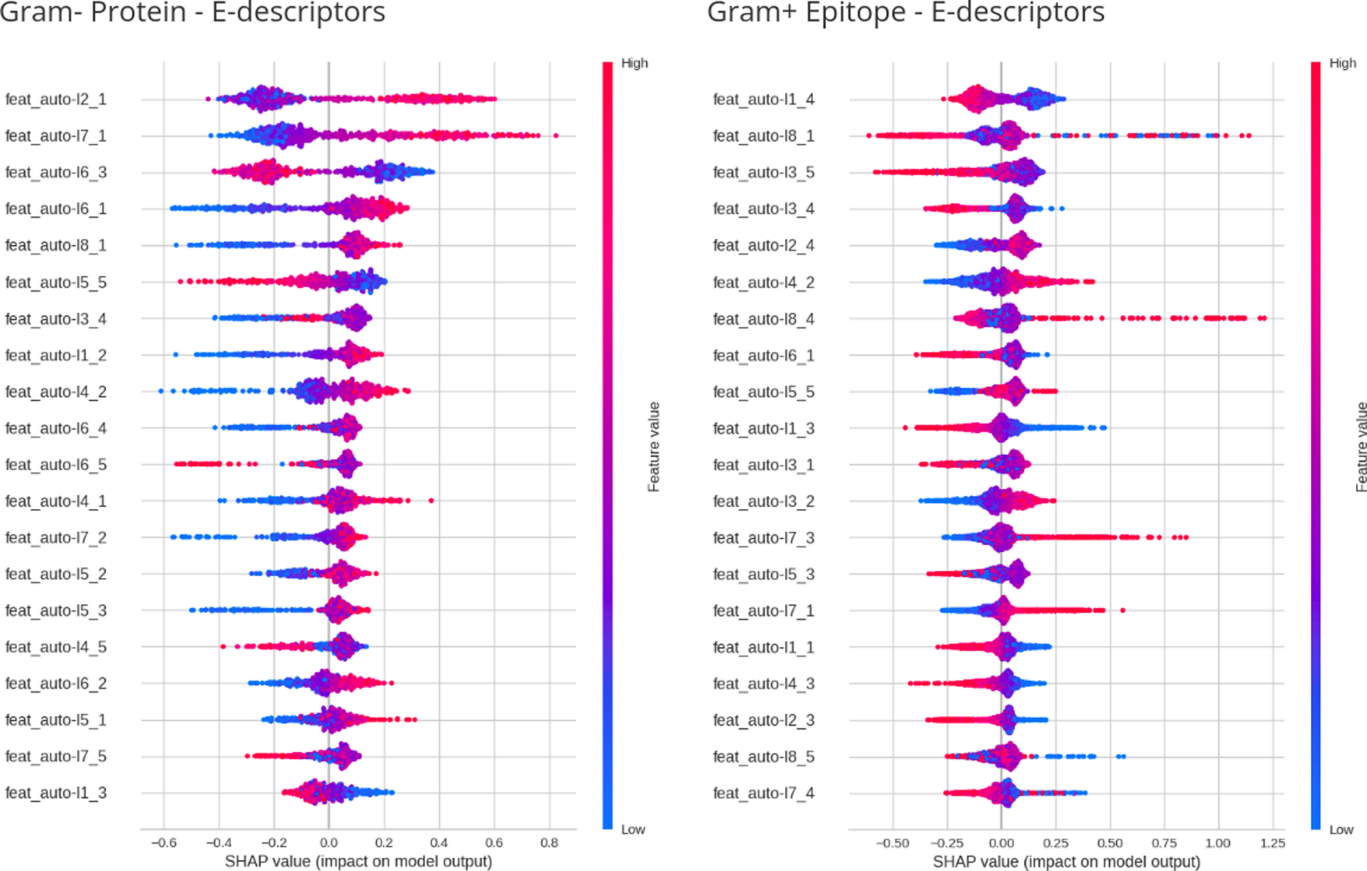
SHAP analysis plots for the best-ranked models for the protein (Gram-negative) and epitope (Gram-positive) sequence types derived from the e-descriptors feature extraction method. The plots show the distribution of the values of each feature and the contribution of their values intensity (high or low) to the model.

To track the interpretability^51^ of the models derived from feature selection upon merging and combining features derived from auto and cross modes for distinct lag lengths, we also provide summary plots concerning the impact of feature values on the model output (based on SHAP method^22,38^). After the training phase with these eight data sets, 16 summary SHAP plots were generated for these two feature extraction methods. We chose one representative plot for each type of sequence (protein and epitope) (Figure 5) from the general ranked list across all 24 models. The selected models were derived from the e-descriptors feature extraction method, and the training data sets for protein and epitope were, respectively, Gram-negative and Gram-positive. In the protein representative plot, the Gram-negative model obtained an ROC-AUC of 76.45% and MCC of 39.81%, while the choice representing the epitope obtained an ROC-AUC of 73.34% and MCC of 33.63%. Figure 5 shows that, despite sharing the exact origin in features (auto mode with identical lag values), these two models exhibit a disjoint distribution of values. In the Gram-negative protein data set, numerous samples with low values negatively impact the model. In contrast, a less condensed trace of high values is observed as negative in the Gram-positive epitope data set.

The domain applicability analysis was performed for all combinations of data sets from both sequence types (protein and epitope). Twenty-five were generated from epitope data sets (hla, bcipep, allgram_epitope, gram-_epitope, and gram+_epitope) and nine from protein (allgram_protein, gram-_protein, and gram+_protein). We set the feature extraction method for this analysis as the one derived from the ESM-2 model embeddings since the results, as mentioned above, showed that it improved most of the metrics. This information is provided in Supplementary Table 5.

As shown in Supplementary Figure 3, the recovery and model evaluation metrics satisfied the assumptions for both sequence types, with test samples derived from the same data set achieving approximately 96% in ROC-AUC and 82% in general neighbor recovery. For protein models, the lowest ROC-AUC values were observed in the (gram-_protein, gram+_protein) and (gram+_protein, gram-_protein) train-test combinations, at 57.72 and 57.76%, respectively, with general recovery dropping to 51.1 and 50.9%. For all other protein model combinations, recovery values exceeded 60%, along with corresponding improvements in ROC-AUC values. Notably, combinations where the allgram_protein data set was used for training consistently achieved ROC-AUC values above 80% across all test data sets.

Interestingly, in the epitope models, the experiments containing the data sets derived from the Protegen data augmentation showed the highest recovery and ROC-AUC values, especially the allgram_epitope cases that obtained 82 and 96% for these metrics. In all combinations in which HLA and Bcipep were either the training or the test data sets, the recovery, and AUC values achieved more than 90% only when the test samples were from themselves; otherwise, the AUC values ranged from 44 to 55%, and the general recovery from 37 to 61%. These results show that the protein models are more reliable than most epitope models, and among the epitope models, the most reliable ones involve data sets derived from Protegen data augmentation. They could generalize the learning and the applicability for other data sets of the same type.

### 3.2 Application of PAPreC Models on IEDB Curated Data

We tested the models generated with the training data sets according to the epitopes and protein contexts. In the epitope context, we also measured the performance of the subgroups derived from T and B cell assays. Based on the information provided by the IEDB, including epitope identifiers, sequences, and protein identifiers, we filtered the valid epitopes according to the restrictions imposed by the external tools employed for comparison with the PAPreC-generated models. We only included epitopes with a sequence length of at least six valid amino acids and those belonging to UniProt-validated protein entries. There were two cases where the sequences contained an uncommon amino acid named Selenocysteine.^52^ These filters were applied because VaxiJen only evaluates sequences with more than five amino acids, and Vaxign-ML cannot process nonstandard amino acids. Table 3 shows the main characteristics of the input data sets that were evaluated in this section. The bacteria species with more experimentally validated epitopes are *Enterobacter*, *S. aureus* and *P. aeruginosa*, corresponding to 98% of all epitopes. For *E. faecium*, there were no representatives for the T-cell epitopes test set.

**Table 3:** Number of Samples in Each Test Dataset Extracted and Processed from the IEDB Database

ESKAPE group	all proteins	all epitopes	T-cell epitopes	B-cell epitopes
*Enterobacter*	253	1801	950	852
*S. aureus*	80	999	100	899
*K. pneumoniae*	15	37	11	26
*A. baumannii*	5	18	12	6
*P. aeruginosa*	25	575	345	232
*E. faecium*	2	6	0	6

**Figure 6:**
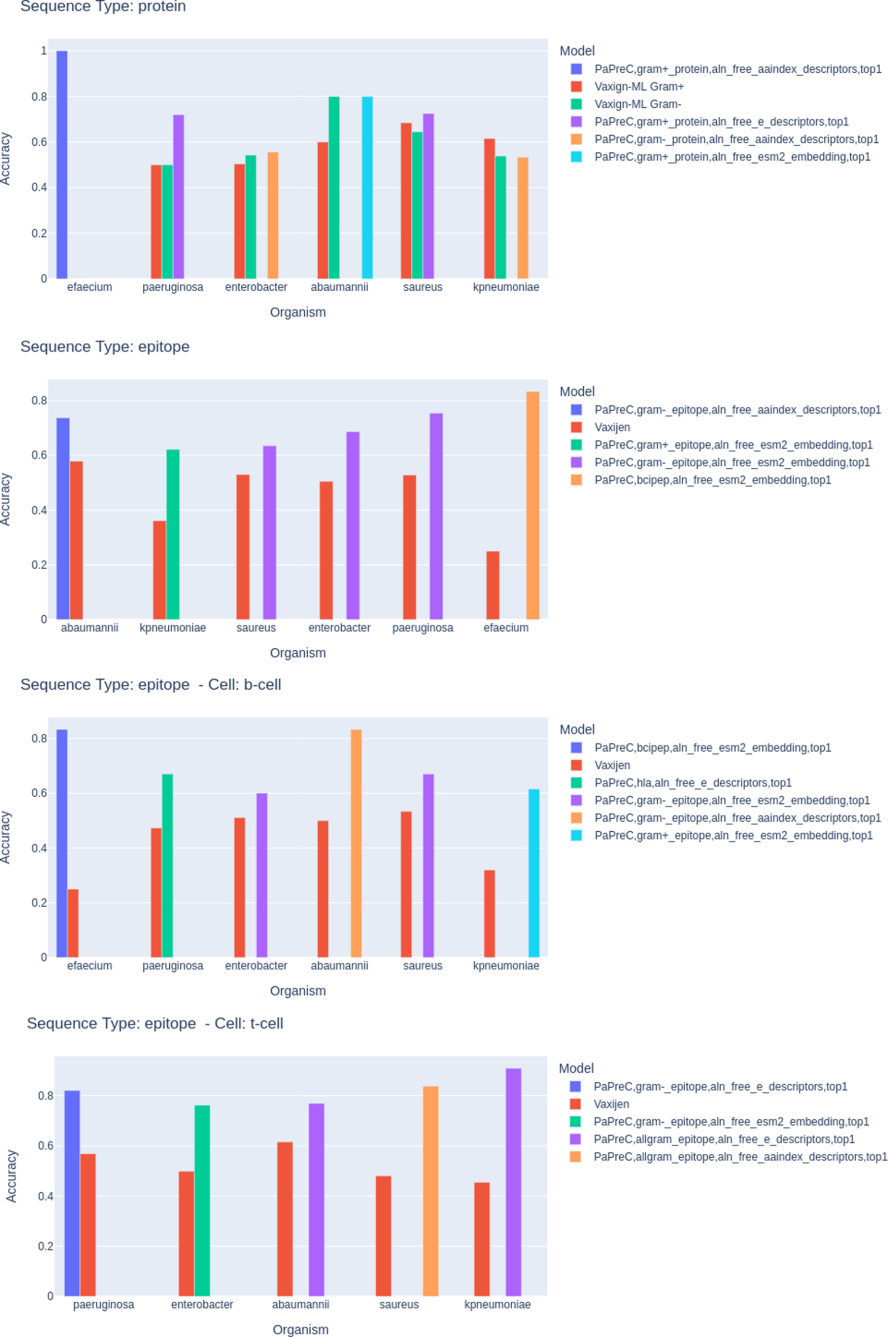
Summary of the accuracy results derived from the analysis of four types of data sets (all proteins, all epitopes, T-cell and B-cell epitopes) for each species of the ESKAPE group (*Enterobacter* spp., *S. aureus*, *K. pneumoniae*, *A. baumannii*, *P. aeruginosa* and *E. faecium*).

Supplementary Table 6 presents the evaluation metrics for the three top-performing models out of the 24 trained configurations (derived from the eight training data sets and three feature extraction methods) alongside results from external tools (VaxiJen or Vaxign-ML). These evaluations are applied to four different data set types across the six species of the ESKAPE group. By employing the originally trained models without retraining or fine-tuning, we can directly assess the generalizability and robustness of the models across diverse bacterial species and data conditions. In the Vaxign-ML results, we tested the protein data sets using the Gram-positive and the Gram-negative variations. In summary, as depicted in Figure 6, The PAPreC models outperform the published methods for antigenicity in almost all 24 data sets, except for the data sets belonging to proteins, where our best model is tied at 80% in *A. baumannii* and has a difference of less than 5% in relation to the Vaxign-ML Gram-positive model. Interestingly, in this last case, the *K. pneumoniae* is a Gram-negative type of bacteria, and their model trained for the Gram-positive type obtained a better accuracy value (about 60%). Notably, the epitope data sets derived from the augmentation strategy obtained the highest values of accuracy (above 80%) in almost all cases, apart from the epitope data set in *E. faecium* and the t-cell epitopes in *P. aeruginosa*. In these cases, the models derived from the Bcipep and HLA training data sets. As observed in the training results from the previous section, the models built from the ESM-2 model embeddings had improved the evaluation metrics, and in these test sets at least one model in each type of data set was derived using this feature extraction strategy.

### 3.3 Model Application on New *P. aeruginosa* and *S. aureus* Antigenicity for MHC Class II Epitopes and Proteins

We submitted 6518 *P. aeruginosa* proteins and 2686 *S. aureus* proteins for class II epitope prediction. Upon submission to epitope prediction by the NetMHCPanII tool,^43^ these proteins generated 433,483 (*P. aeruginosa*) and 207,278 epitopes (*S. aureus*).

We analyzed the selected epitopes using an adapted version of the Epitopes Curator pipeline (Epicurator).^53^Table 4 summarizes the epitopes that were removed by each curation step. We activated the binding affinity prediction information to be calculated by the epitope predictor and selected only those with solid binding scores to the MHC alleles. We selected only those with a 2% or less percentile rank to ensure this restriction. This filter removed 68.5% of the predicted epitopes for *P. aeruginosa* and 73.86% of those from *S. aureus*.

**Table 4:** Number of Epitopes Excluded in Each Curation Step^a^

step/organism	*P. aeruginosa*	*S. aureus*
rank percentile binding affinity	296,989	153,116
overlapping protegen proteins	146	259
overlapping human proteins	2	0
total	297,137	153,375

a The first filter removes epitopes with a binding affinity percentile ranking below 2%. The second filter removes peptides already present in the protein sequences listed in the Protegen database. The final filtering step excludes epitopes with 100% identity and coverage to human protein sequences.

Significant epitopes were already part of curated immunogenic bacterial proteins in Protegen, mainly for *S. aureus* (259). In *P. aeruginosa*, the hits matched 26 distinct proteins, while for *S. aureus* matched 33 proteins. However, most of the proteins in the *S. aureus* set belong to other strains of *S. aureus*, such as A8115 and Newman, and only one protein was from a different species (*Streptococcus agalactiae*). Only one of the proteins was from *Pseudomonas aeruginosa* in the *P. aeruginosa* protein set. Some other species found were *Shigella flexneri* five str. 8401, *Helicobacter pylori*, *Escherichia coliETEC H10407*, and *Chlamydia muridarum* str. Nigg and *Actinobacillus pleuropneumoniae*. Finally, since 40% of the human mitochondrial proteins are of bacterial origin, we encountered a few epitopes corresponding to human mitochondrial isoforms.^54^ The 2 epitopes excluded in this step were the epitope “AAVGASRAAVDAGFV”, which matched 12 proteins, and “GFEIAQGRLGPGRIH” that matched 2 human proteins.

At the end of the curation step, 136,339 epitopes and 6425 proteins remained for *P. aeruginosa*, and 53,895 epitopes from 2643 proteins for *S. aureus*, composed the test set for the antigenicity prediction comparison by the PAPreC pipeline (Supplementary Table 7). The top-ranked models of each type (protein and epitope) from the previous section analysis formed the committee to evaluate the new epitopes and their proteins, assuring that these models have obtained the best MCC value for each combination of training data set and feature extraction method. We chose the final list of antigenic proteins and epitopes if at least 50% of the models agreed with the same class. For *P. aeruginosa*, 30,719 epitopes and 1415 unique proteins were classified as antigenic by VaxiJen 2.0 server and Vaxign-ML tool, respectively. The results for *S. aureus* were 10,808 epitopes and 867 unique proteins categorized as antigens. As detailed in Supplementary Table 8, for both species, the agreement of the PAPreC models concerning the VaxiJen and Vaxign-ML tools reached an average of 62.8% (*P. aeruginosa*) and 57.8% (*S. aureus*), achieving more than 70% only in the protein sets in three models for *P. aeruginosa* and one for *S. aureus*. Interestingly the highest agreement was achieved mainly for the training data sets derived from Protegen augmentation and using the ESM-2 model embeddings as a feature extraction method.

According to the prediction results summarized in Table 5, many epitopes and proteins were selected as positive. Our criteria to elect the positive cases was at least one model targeting the sequence type voting for the positive class (1). To account for this, besides the summary table of predictions according to the models, we provide an auxiliary table with the agreement percentage across the models and the names of the models that voted for the positive class to guide the user of the PAPreC results.

**Table 5:** Summary of Predicted Positive and Negative Cases by Organism and Sequence Type

organism	sequence type	qty. positive	qty. negative
*P. aeruginosa*	protein	5012	1412
*P. aeruginosa*	epitope	135,845	494
*S. aureus*	protein	1951	692
*S. aureus*	epitope	53,806	89

Following the evaluation in line with the recovery from the Protegen positive set augmented with IEDB-validated data, we evaluated whether the positively predicted proteins obtained a similarity of at least 80% for proteins and 100% for epitopes with the positive set. The Supplementary Table 9 shows 12 protein hits for *P. aeruginosa* and 19 for *S. aureus*, while the number of hits for epitopes was 268 and 81, respectively. All hits within the positive protein set were correctly classified as positive by PAPreC models. Considering all hits from both species, the average probability score was approximately 68% for proteins and 58% for epitopes. Interestingly, there were two cases among these hits that the proteins also shared Pfam families; the protein identified by pa4408_04429 obtained the highest score (81.8%) and matched the Exotoxin-A^55^ family, which is present in more than 90% of the proteins present in IEDB for *P. aeruginosa*. Still, in *P. aeruginosa*, we found a hit (pa4408_04681) that shared the OmpA domain with the positive set that obtained a score of 76.11%. This domain has been studied as a potential target for vaccines for *K. pneumoniae*.^56^ These two highlighted domains received the highest probability scores. All protein models classified The Exotoxin-A domain as positive, while the protein with the OmpA domain was classified as positive by 88% of the models. Additionally, 31 proteins demonstrated at least 80% sequence similarity with the positive protein set, and 30 of these shared at least one domain with the reference proteins.

We prepared a list (Supplementary Table 10) containing the ranked list of positively predicted epitopes and proteins. In this list, we also annotated the Pfam families predicted for the proteins. Of the 6963 proteins, 765 had no hits in the Pfam database for families.

Among these proteins, the previously mentioned Exotoxin A achieved the highest score (0.81) for *Pseudomonas aeruginosa*. Known as the bacterium’s most toxic virulence factor, Exotoxin A disrupts host protein synthesis via ADP-ribosylation. DNA vaccines encoding a truncated version of Exotoxin A have shown promising results, inducing specific immune responses and providing protective immunity in mice against lethal toxin doses.^57^ Fusion constructs with Exotoxin A have also yielded favorable outcomes, enhancing its potential as a vaccine candidate.^58,59^

Another promising candidate with a high score is PopB, a type III secretion system protein. It has demonstrated potential in vaccine formulations. When paired with its chaperone protein PcrH and administered with adjuvants, PopB stimulates IL-17-mediated Th17 responses, providing protection against *P. aeruginosa* lung infections. Encapsulating PopB/PcrH in PLGA nanoparticles further enhances these protective Th17 responses, reducing bacterial counts in the lungs and improving survival rates in mice.^60^

Then, we selected the proteins with antigenicity results according to PAPreC in *P. aeruginosa* and *S. aureus*, and we highlight some of these below with results already published in the literature.

In *S. aureus*, we can highlight proteins on our list that have already been identified as promising targets for vaccine development against this important pathogen. In this context, the Isd proteins of the iron acquisition system are particularly noteworthy. The Isd system, one of the most relevant for the pathogenesis of *S. aureus*, is composed of nine proteins, four of which (IsdA, IsdH, IsdE, and IsdC) are present in the PAPreC prediction, including the IsdH (sabb_01856) with the highest score of our prediction (0.83).^61^ IsdE (sabb_06149) with the score of 0.61, elicited a strong immune response through various experimental methods and demonstrated as a promising target for vaccine development against methicillin-resistant *S. aureus* (MRSA).^62^ The role of cell wall-anchored proteins as vaccine antigens in combating *S. aureus* infections has garnered significant attention, with a particular focus on the immunogenic potential of clumping factors (Clfs) A and B, encoded by the *clfA* and *clfB* genes, respectively. The proteins ClfA and ClfB promote adhesion to fibrinogen and play essential roles in the pathogenesis and virulence of *S. aureus* by, for example, activating platelet aggregation and inhibiting phagocytosis.^63−65^ In addition to facilitating nasal colonization of *S. aureus* through its interaction with the ligands loricrin and cytokeratin 10, ClfB plays a crucial role in skin and soft tissue infections (SSTIs) (Lacey et al.). A model vaccine formulated with ClfB in combination with the adjuvant CpG induced both protective humoral and cellular immune responses, demonstrating that ClfB is a promising antigen candidate for inclusion in vaccines targeting *S. aureus* SSTIs.^66^ A previous study had already shown the potential of ClfB as an attractive target for vaccines aimed at reducing nasal colonization of *S. aureus* in humans.^67^ Beyond its importance in the virulence of *S. aureus*, ClfA with the second highest score (0.8) in our prediction has been proposed as a promising target for inclusion in multivalent vaccines by numerous studies due to its ability to induce a robust immune response.^68,69^ Indeed, Pfizer and other biopharmaceutical companies are investigating in clinical trials ClfA-based vaccines as a potential way to prevent *S. aureus* infections. For instance, SA4Ag, a 4-antigen *S. aureus* vaccine, has been designed to target three key virulence mechanisms associated with staphylococcal disease: immune evasion (capsule), adherence to host molecules and immune evasion (ClfA) and nutrient acquisition (manganese transporter C - MntC).^68,70^ The SA4Ag aims to prevent invasive *S. aureus* diseases, demonstrating the success of ClfA as a pivotal component in vaccine development strategies.^71^ This finding highlights the importance of these cell surface proteins as promising targets for vaccines against *S. aureus* infections.^72^

## 4 Discussion

The study presented here offers valuable insights into the performance and effectiveness of antigenicity prediction models. We systematically evaluated factors such as data set composition, feature extraction methods, and classifier selection to compare different models on the antigenicity task. Our primary goal in developing the PAPreC workflow was to enable structured adaptation and personalization, allowing researchers to systematically screen models for antigenicity prediction in both peptide and protein sequences, analyze new data, and refine the evaluation of trained models’ applicability.

Our study focused on epitopes and whole protein sequences, utilizing the Bcipep, compiled HLA and Protegen protein sequence data sets. We proposed a data augmentation strategy to update the Protegen positive set using IEDB data and generate negative samples. A two-layer ranking system was implemented, first ranking classifiers by the user-configured metric (default MCC) and selecting the best classifier to represent each data set-feature extraction combination. Our pipeline, available on GitHub with detailed instructions for running and extending tasks, ensures flexibility, transparency, and model evaluation. The intention is not to achieve the best predictions but to analyze the behavior of top models compared to published predictors and IEDB reference data.

We aimed to provide a ‘black-box opening’ approach in which users can adapt the workflow to their data sets, research objectives, and computational resources, thereby extending the primary architecture as needed. To achieve this goal, we chose Nextflow as our workflow management system, benefiting from its broad adoption and flexibility in bioinformatics. Using this framework, we successfully ran the entire pipeline on a standard laptop without needing high-performance computing. The most computationally intensive steps—training, evaluation, and data parsing—took under 4 h, primarily due to comparisons against the human proteome and its isoforms.

Recent studies^73−75^ have employed open-source workflow management systems to streamline peptide characterization, often incorporating AutoML frameworks and tools like KNIME. These approaches facilitate model generation, classifier optimization, and model interpretability. Here, we leverage PyCaret’s AutoML capabilities in our workflow but extend the screening process to earlier stages, including training data set assembly and feature extraction method selection.

Regarding model performance, we observed that the choice of feature extraction method had a significant impact. Feature selection improved performance metrics for both data sets and methodologies, with Random Forest and Extra Trees consistently outperforming Gradient Boosting and the other classifiers in the set. The performance evaluation results demonstrated that embeddings derived from the ESM-2 autoencoder model enhanced performance, particularly for training data sets based on whole proteins. Although larger ESM-2 models with 30 or 33 layers improve accuracy and reduce feature dimensionality to approximately 1280,^74,76−78^ we opted for the lighter configuration to ensure computational feasibility. To capture meaningful sequence features without overburdening memory, we truncated embeddings to 2048 dimensions, as recommended by recent studies.^32,35^ This trade-off between model complexity and computational efficiency makes our workflow more accessible and adaptable.

Fluctuations in evaluation metric outcomes during the training phase were observed, attributed to variations in training data distributions, epitope types, and the complexity of antigenicity prediction. PAPreC helps identify conditions for model optimization, guiding improvements such as enhanced training data, refined feature extraction, and incorporation of additional biological knowledge.

The feature selection strategy, ranking features from all method variations in the top 1 over 80% of the time, proved effective in reducing dimensionality and complexity. To ensure transparency, we included explainability tools like feature importance analysis and a SHAP summary. These revealed distinct value patterns for epitope and protein features, helping differentiate cases. The applicability domain analysis showed robust model confidence and evaluation metrics (accuracy, ROC-AUC) above 70%, especially for protein models, with the best results from Protegen augmentation.

We compared our models with Vaxign-ML and VaxiJen 2.0 in two scenarios: using validated IEDB data for the ESKAPE group and a new set predicted from a case study of selected *S. aureus* and *P. aeruginosa* proteomes. In the first scenario, our models outperformed the comparison tools in over 95% of tests, demonstrating their robustness. In the second one, with new peptides and proteins, our predictions showed 60% agreement, indicating consistency with IEDB comparison results. Our workflow is adaptable to any training data set using positive and negative antigenic samples.

Additionally, our study evaluated the generalization capability of the models in predicting antigenicity across different types of bacterial proteins. The results demonstrated that our models trained on Gram-positive, Gram-negative, and mixed samples achieved high evaluation metric scores. This finding indicates that separate models for different Gram types, as proposed by Vaxign-ML, are unnecessary. In this study, we evaluated the bacterial data sets, and there were exclusively T-cell or B-cell epitopes in the peptide data sets. However, Zhou et al. demonstrated that transfer learning can successfully predict virus antigenicity when applied to models trained on diverse data sources.^79^

We applied the Epitopes Curator pipeline (Epicurator) to the proteomes of *P. aeruginosa* strain 14182 and *S. aureus* Bmb9393 to validate our predicted epitopes. We analyzed the predicted positive epitopes and their similarity with the positive set, identifying hits over 90%. Additionally, we recovered proteins and epitopes excluded during filtering, identifying key antigenic Pfam domains with high probability scores relevant to vaccine development.

The models trained with the HLA data set achieved the highest agreement with the VaxiJen 2.0 server, reaching 76.82% for *P. aeruginosa* and 80.82% for *S. aureus*. The best result for the Bcipep data set came from the first predictor with a lag of five, trained by Stochastic Gradient Descent. These results highlight the data set’s generalization capability, particularly for T-cell epitopes.

In light of the case study concerning the antigenicity prediction of epitopes and proteins in *P. aeruginosa* strain 14182 and *S. aureus* strain Bmb9393, the outcomes exhibiting consensus between the two presently prevalent tools have enabled us to prioritize a notable subset of proteins. These identified antigenic epitopes and proteins stand out as promising candidates for prospective investigations, offering a valuable avenue for further analysis and exploration.

## 5 Conclusions

The Pipeline for Antigenicity Prediction Comparison (PAPreC) offers a comprehensive framework for evaluating bacterial protein and epitope sequences, incorporating various feature selection strategies and classifiers to optimize predictions. Accessible via GitHub (https://github.com/YasCoMa/paprec_nx_workflow), it enables extensions with additional data sets or feature extraction methods. Our study highlights the importance of feature selection and demonstrates the improved performance of models using the ESM-2 autoencoder. Compared to established tools, PAPreC outperforms predictions for ESKAPE pathogens, particularly with IEDB data sets. We also predicted novel epitopes using Protegen, advancing our understanding of antigenicity and refining future vaccine and therapy development prediction models.

## Supporting Information Available

Figure S1: panel illustrating the metrics obtained in each combination of training data set and feature extraction method separated by metric: Accuracy (A), ROC-AUC (B), F1 (C), and MCC (D); Figure S2: panel illustrating the metrics obtained in each combination of training data set and classifier separated by metric: Accuracy (A), ROC-AUC (B), F1 (C), and MCC (D); and Figure S3: heat maps illustrating the performance metrics (ROC-AUC and general coverage) of the applicability domain analysis for all combinations of training and test data sets related to epitopes and proteins (PDF)
Table S1: table containing information about the epitope data retrieved from the IEDB database, with the PubMed IDs related to the assay that validated these epitopes, also reporting their source organism, protein, the Gram stain (+ or −) and the protein sequence. The table also shows the qualitative measure reported in the assay results (positive or negative); Table S2: negative set report concerning all the protein sequences of the proteomes recovered and the number of epitopes and proteins in the positive set that had hits on these sequences, excluding them as negative sample candidates. The table contains the length of the epitope sequences in the hits, and only the sequences with zero hits in both cases were considered negative candidates; Table S3: prediction results for each training data set, the alignment-free method used for feature extraction, method variation, and the classifier selected for the top-ranked models based on the MCC metric across 24 combinations are presented. The table provides the corresponding values for accuracy, ROC-AUC, precision, recall, F1-score, kappa, and MCC; Table S4: results from the feature importance analysis for each training data set and feature extraction method. The table shows the number of features before and after selection and the importance values for each selected feature across the various combinations; Table S5: table with results of the applicability domain analysis in an all-against-all mode comparison among the training data sets. The data comprises the similarity metrics used in our assessment: the values for the general, positive, and negative coverage of the inliers related to the test data set, and the evaluation metrics (accuracy, precision, recall, F1, ROC-AUC, and MCC) concerning the retrieved inliers; Table S6: results of the evaluation metrics showing the performance of PAPreC models and external established tools (VaxiJen and Vaxign-ML) in the derived data sets from validated data in IEDB for the ESKAPE group. The table contains the type of sequence, the Gram stain, the organism’s short name, the cell type, the model identifier (Origin column), and the values obtained for accuracy, ROC-AUC, precision, recall, F1, kappa, and MCC; Table S7: epitopes and proteins selected in the first step of the filtering process applied by PAPreC, based on raw input derived from the NetMHCPanII output. The table contains the organism’s short name, the epitope identifier and sequence, and the protein identifier from which the epitope was extracted; Table S8: report of agreement among the predictions released by PAPreC models and the published tools for antigenicity (Vaxign-ML and VaxiJen). The table contains the sequence type, the organism’s short name, the comparison source (VaxiJen or Vaxign-ML tool), the combination (training data set/method), and the values obtained for accuracy, ROC-AUC, precision, recall, F1, kappa, and MCC; Table S9: list of proteins positively predicted by PAPreC that obtained a 90% sequence similarity with the Protegen set of proteins. The table contains the sequence type, the organism’s short name, the identifier and sequence, score, classification (1 or 0), and the agreement among the PAPreCmodel of the respective sequence type; and Table S10: list of epitopes and proteins positively predicted by our models, including the annotated Pfam families assigned to each protein. The table contains the sequence type, the organism’s short name, the identifier, sequence, score, and the agreement among the PAPreC model of the respective sequence type. The table also reports the annotated Pfam identifiers and their protein names (XLSX)
