# Supplementary material for: PAPreC: A Pipeline for Antigenicity Prediction Comparison Methods across Bacteria
Source: ACS Omega. 2025 Feb 3;10(6):5415–29. doi: 10.1021/acsomega.4c07147 (PMC11840615; doi:10.1021/acsomega.4c07147)

Supporting information for the article "PAPreC: A Pipeline for Antigenicity Prediction Comparison Methods Across Bacteria"

Yasmmin C. Martins[1,2], Maiana O. Cerqueira e Costa[1], Miranda C. Palumbo[2], Dario F. Do Porto[2], Fábio L. Custódio[3], Raphael Trevizani[4], and Marisa Fabiana Nicolás[1*]

[1]Bioinformatics Laboratory, National Laboratory for Scientific Computing, Av. Getúlio Vargas 333, 25651-075, Petrópolis-Brazil
[2]Department of biological chemistry, Faculty of Exact and Natural Sciences, University of Buenos Aires - UBA, Av. Int. Cantilo, C1428, Buenos Aires-Argentina
[3]Department of Computational Mechanics, National Laboratory for Scientific Computing, Av. Getúlio Vargas 333, 25651-075, Petrópolis-Brazil
[4]Biotechnology, Oswaldo Cruz Foundation - Fiocruz, Street São José S/N, 61760-000, Eusébio-Brazil
[*]Address correspondence to: marisa@lncc.br

1 Figure S1

Panel illustrating the metrics obtained in each combination of training DB and feature extraction method separated by metric: Accuracy (A), ROC-AUC (B), F1 (C) and MCC (D).

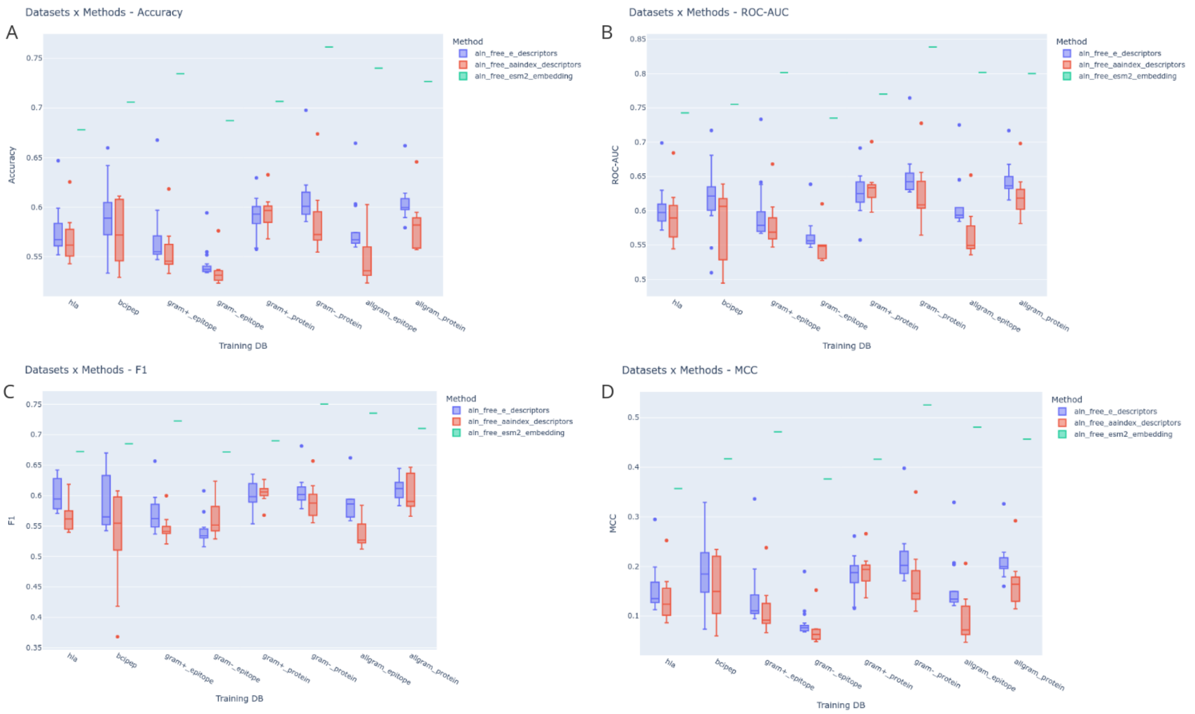

2 Figure S2

Panel illustrating the metrics obtained in each combination of training DB and classifier separated by metric: Accuracy (A), ROC-AUC (B), F1 (C) and MCC (D).

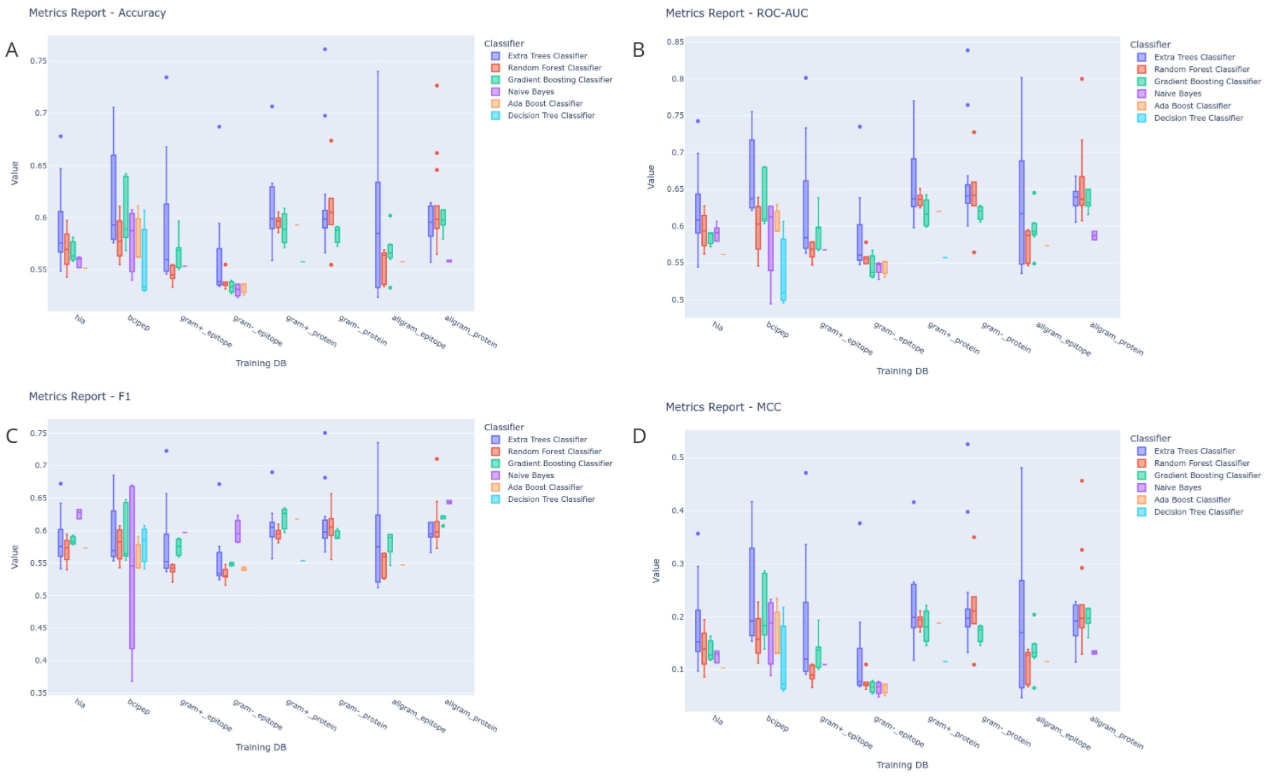

3 Figure S3

Heat maps illustrating the performance metrics (ROC-AUC and general coverage) of the applicability domain analysis for all combinations of training and test datasets related to epitopes and proteins.

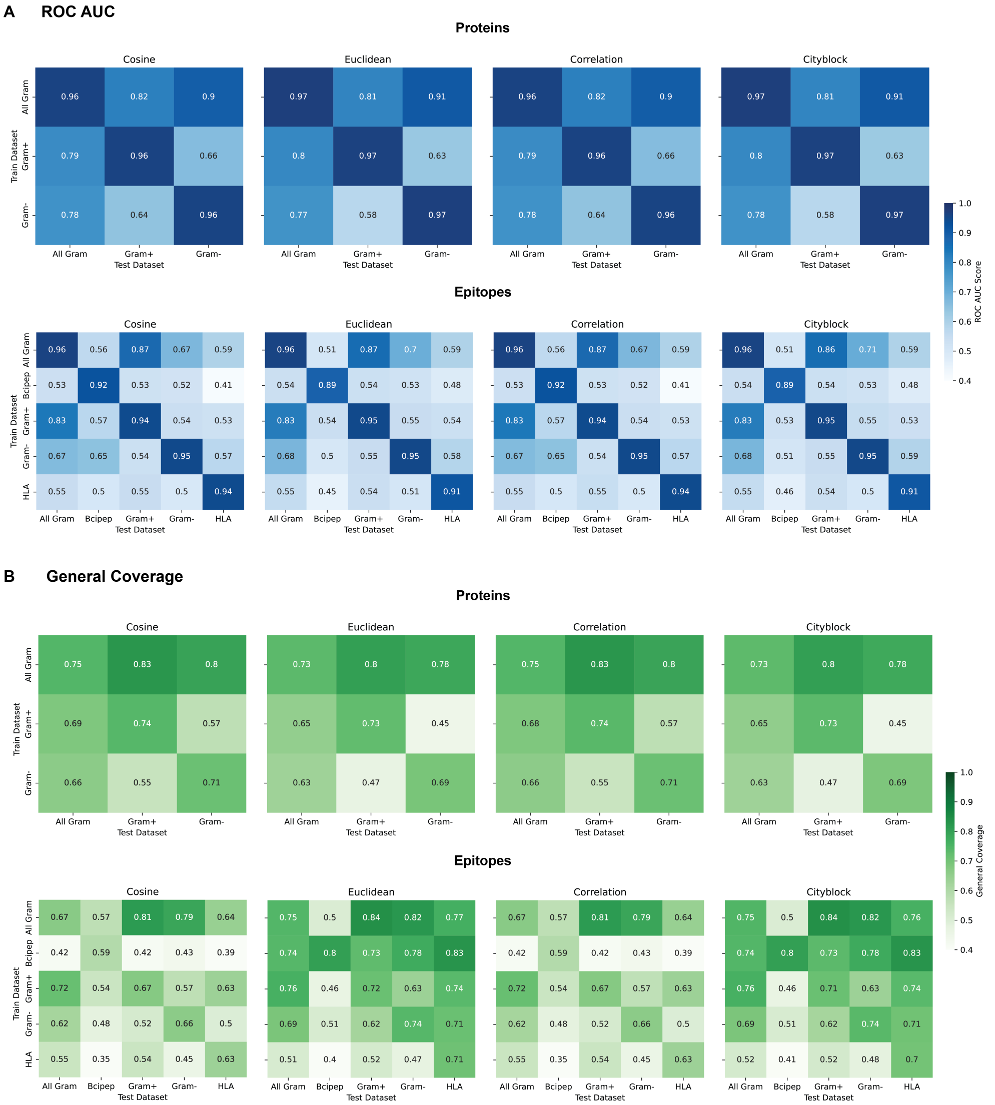

Supplement: Supplementary file 1 — ao4c07147_si_001.pdf [file ao4c07147_si_001.pdf]
